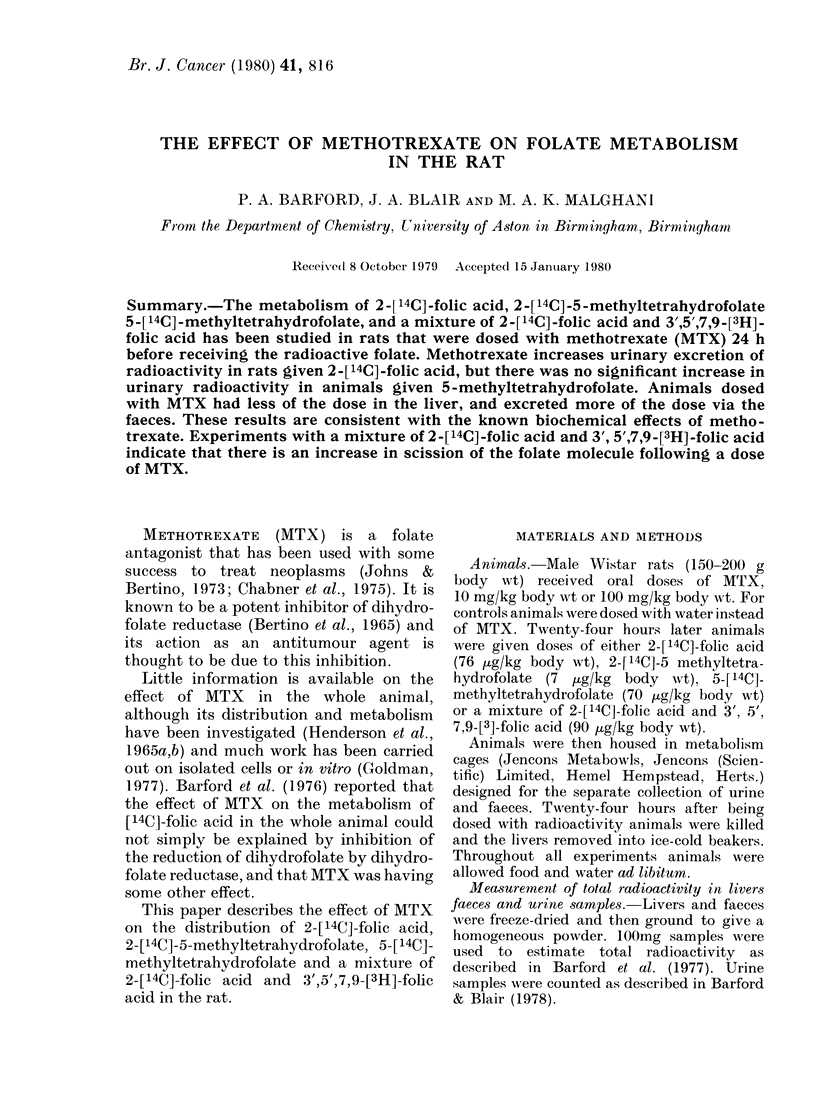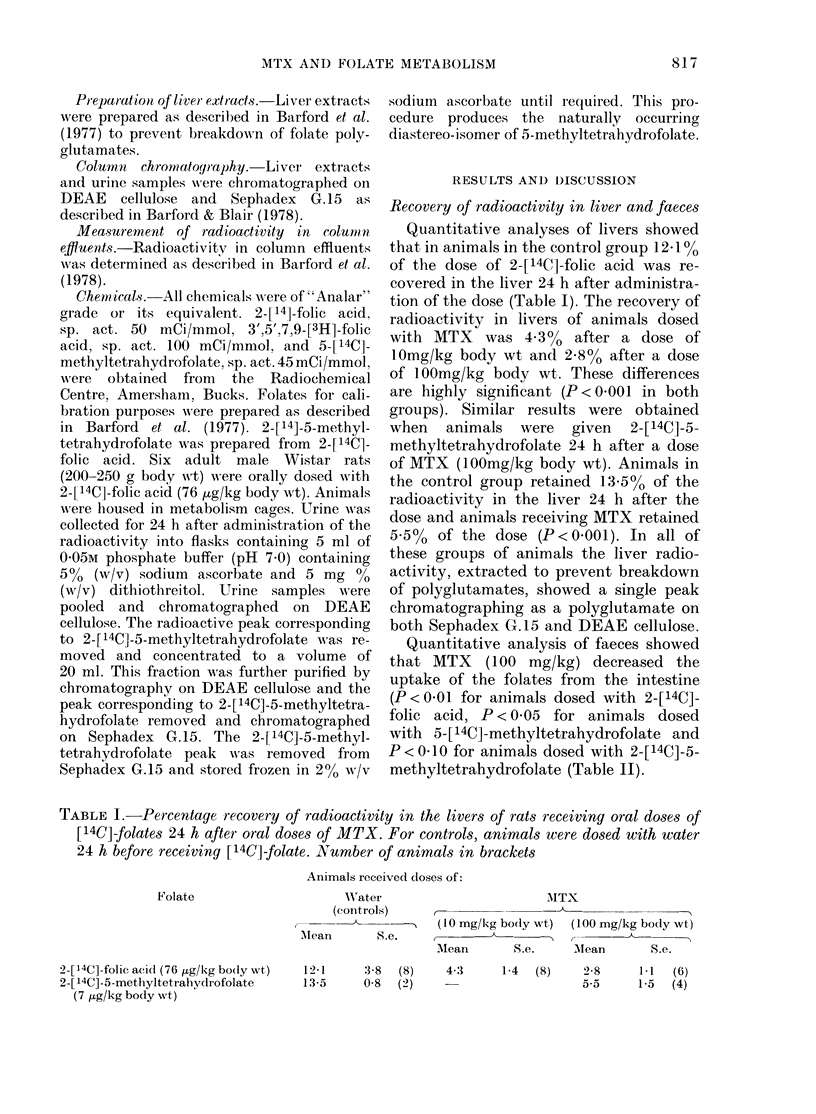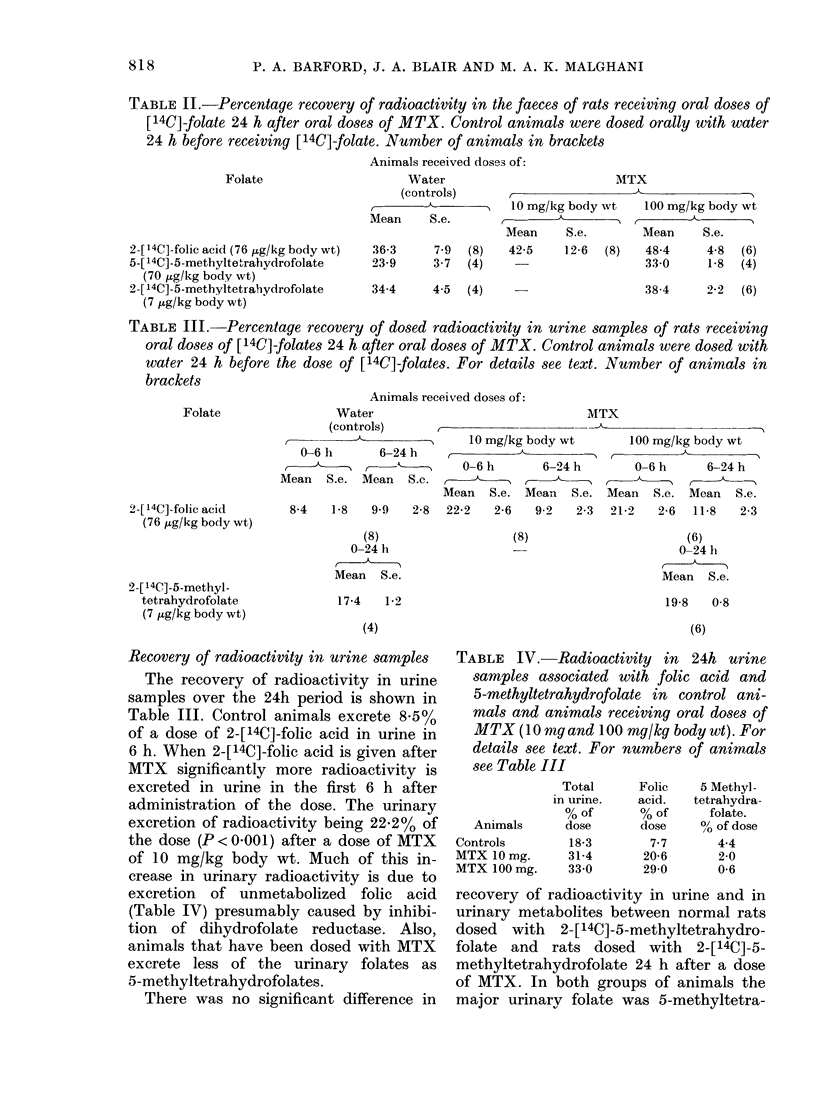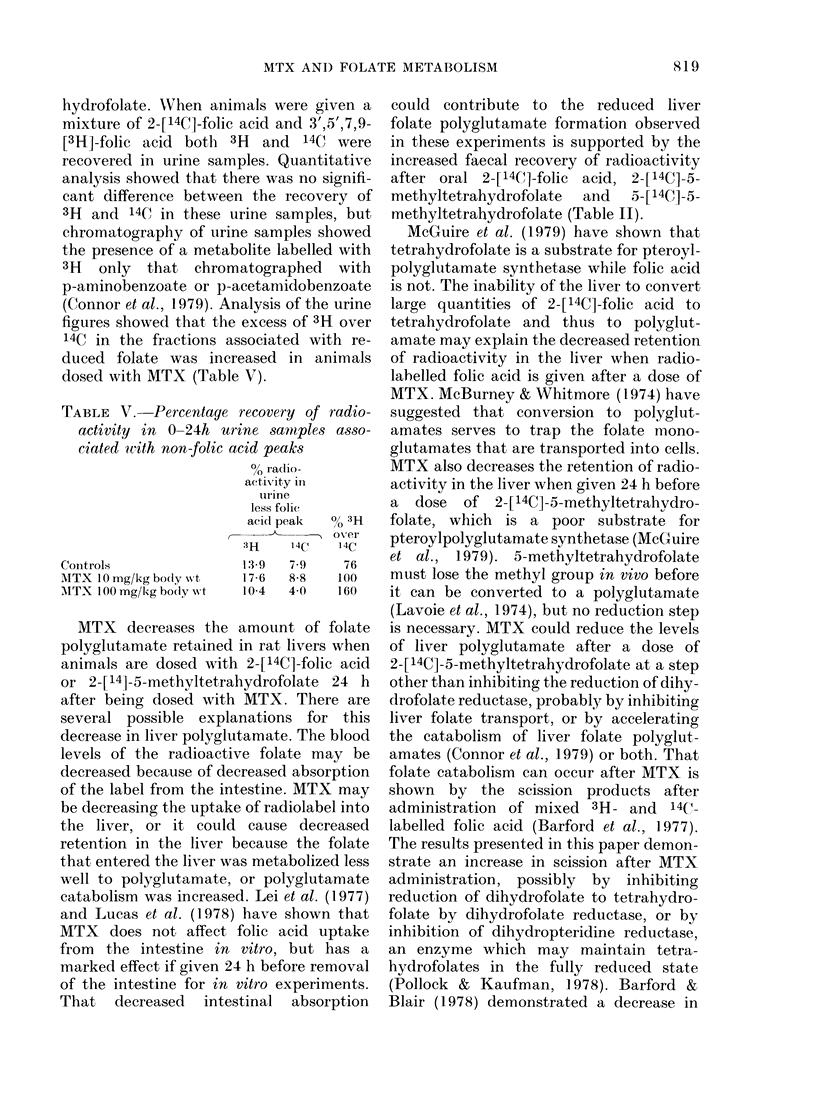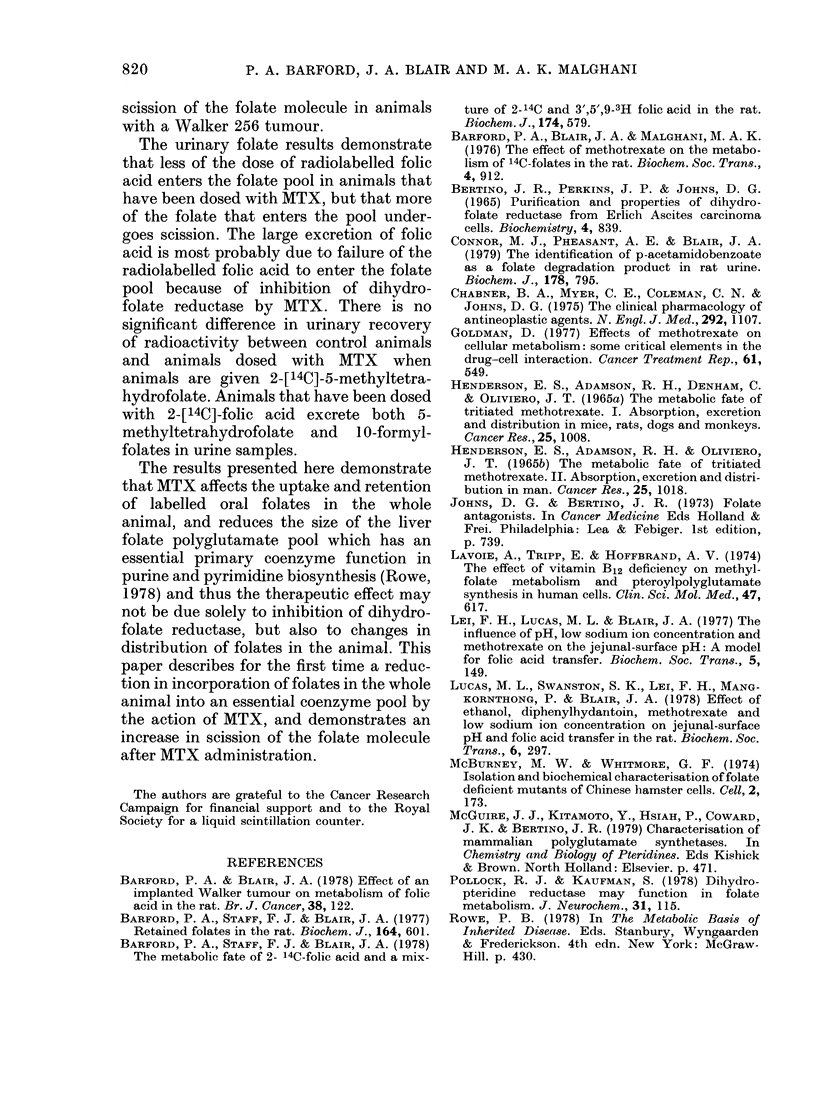# The effect of methotrexate on folate metabolism in the rat.

**DOI:** 10.1038/bjc.1980.146

**Published:** 1980-05

**Authors:** P. A. Barford, J. A. Blair, M. A. Malghani

## Abstract

The metabolism of 2-[14C]-folic acid, 2-[14C]-5-methyltetrahydrofolate 5-[14C]-methyltetrahydrofolate, and a mixture of 2-[14C]-folic acid and 3',5',7,9-[3H]-folic acid has been studied in rats that were dosed with methotrexate (MTX) 24 h before receiving the radioactive folate. Methotrexate increases urinary excretion of radioactivity in rats given 2-[14C]-folic acid, but there was no significant increase in urinary radioactivity in animals given 5-methyltetrahydrofolate. Animals dosed with MTX had less of the dose in the liver, and excreted more of the dose via the faeces. These results are consistent with the known biochemical effects of methotrexate. Experiments with a mixture of 2-[14C]-folic acid and 3',5',7,9-[3H]-folic acid indicate that there is an increase in scission of the folate molecule following a dose of MTX.


*Br. J. Cancer* (1980) **41**, 816

### THE EFFECT OF METHOTREXATE ON FOLATE METABOLISM IN THE RAT

P. A. BARFORD, J. A. BLAIR AND M. A. K. MALGHANI

*From the Department of Chemistry, University of Aston in Birmingham, Birmingham*

Received 8 October 1979 Accepted 15 January 1980

**Summary.—The metabolism of 2-[$^{14}$C]-folic acid, 2-[$^{14}$C]-5-methyltetrahydrofolate 5-[$^{14}$C]-methyltetrahydrofolate, and a mixture of 2-[$^{14}$C]-folic acid and 3′,5′,7,9-[$^{3}$H]-folic acid has been studied in rats that were dosed with methotrexate (MTX) 24 h before receiving the radioactive folate. Methotrexate increases urinary excretion of radioactivity in rats given 2-[$^{14}$C]-folic acid, but there was no significant increase in urinary radioactivity in animals given 5-methyltetrahydrofolate. Animals dosed with MTX had less of the dose in the liver, and excreted more of the dose via the faeces. These results are consistent with the known biochemical effects of methotrexate. Experiments with a mixture of 2-[$^{14}$C]-folic acid and 3′, 5′,7,9-[$^{3}$H]-folic acid indicate that there is an increase in scission of the folate molecule following a dose of MTX.**

Methotrexate (MTX) is a folate antagonist that has been used with some success to treat neoplasms (Johns & Bertino, 1973; Chabner *et al.*, 1975). It is known to be a potent inhibitor of dihydrofolate reductase (Bertino *et al.*, 1965) and its action as an antitumour agent is thought to be due to this inhibition.

Little information is available on the effect of MTX in the whole animal, although its distribution and metabolism have been investigated (Henderson *et al.*, 1965*a,b*) and much work has been carried out on isolated cells or *in vitro* (Goldman, 1977). Barford *et al.* (1976) reported that the effect of MTX on the metabolism of [$^{14}$C]-folic acid in the whole animal could not simply be explained by inhibition of the reduction of dihydrofolate by dihydrofolate reductase, and that MTX was having some other effect.

This paper describes the effect of MTX on the distribution of 2-[$^{14}$C]-folic acid, 2-[$^{14}$C]-5-methyltetrahydrofolate, 5-[$^{14}$C]-methyltetrahydrofolate and a mixture of 2-[$^{14}$C]-folic acid and 3′,5′,7,9-[$^{3}$H]-folic acid in the rat.

### MATERIALS AND METHODS

*Animals.*—Male Wistar rats (150–200 g body wt) received oral doses of MTX, 10 mg/kg body wt or 100 mg/kg body wt. For controls animals were dosed with water instead of MTX. Twenty-four hours later animals were given doses of either 2-[$^{14}$C]-folic acid (76 $\mu$g/kg body wt), 2-[$^{14}$C]-5 methyltetrahydrofolate (7 $\mu$g/kg body wt), 5-[$^{14}$C]-methyltetrahydrofolate (70 $\mu$g/kg body wt) or a mixture of 2-[$^{14}$C]-folic acid and 3′, 5′, 7,9-[$^{3}$]-folic acid (90 $\mu$g/kg body wt).

Animals were then housed in metabolism cages (Jencons Metabowls, Jencons (Scientific) Limited, Hemel Hempstead, Herts.) designed for the separate collection of urine and faeces. Twenty-four hours after being dosed with radioactivity animals were killed and the livers removed into ice-cold beakers. Throughout all experiments animals were allowed food and water *ad libitum.*

*Measurement of total radioactivity in livers faeces and urine samples.*—Livers and faeces were freeze-dried and then ground to give a homogeneous powder. 100mg samples were used to estimate total radioactivity as described in Barford *et al.* (1977). Urine samples were counted as described in Barford & Blair (1978).

*Preparation of liver extracts.*—Liver extracts were prepared as described in Barford *et al.* (1977) to prevent breakdown of folate polyglutamates.

*Column chromatography.*—Liver extracts and urine samples were chromatographed on DEAE cellulose and Sephadex G.15 as described in Barford & Blair (1978).

*Measurement of radioactivity in column effluents.*—Radioactivity in column effluents was determined as described in Barford *et al.* (1978).

*Chemicals.*—All chemicals were of "Analar" grade or its equivalent. 2-[14]-folic acid, sp. act. 50 mCi/mmol, 3′,5′,7,9-[$^3$H]-folic acid, sp. act. 100 mCi/mmol, and 5-[$^{14}$C]-methyltetrahydrofolate, sp. act. 45 mCi/mmol, were obtained from the Radiochemical Centre, Amersham, Bucks. Folates for calibration purposes were prepared as described in Barford *et al.* (1977). 2-[14]-5-methyltetrahydrofolate was prepared from 2-[$^{14}$C]-folic acid. Six adult male Wistar rats (200–250 g body wt) were orally dosed with 2-[$^{14}$C]-folic acid (76 μg/kg body wt). Animals were housed in metabolism cages. Urine was collected for 24 h after administration of the radioactivity into flasks containing 5 ml of 0·05M phosphate buffer (pH 7·0) containing 5% (w/v) sodium ascorbate and 5 mg % (w/v) dithiothreitol. Urine samples were pooled and chromatographed on DEAE cellulose. The radioactive peak corresponding to 2-[$^{14}$C]-5-methyltetrahydrofolate was removed and concentrated to a volume of 20 ml. This fraction was further purified by chromatography on DEAE cellulose and the peak corresponding to 2-[$^{14}$C]-5-methyltetrahydrofolate removed and chromatographed on Sephadex G.15. The 2-[$^{14}$C]-5-methyltetrahydrofolate peak was removed from Sephadex G.15 and stored frozen in 2% w/v sodium ascorbate until required. This procedure produces the naturally occurring diastereo-isomer of 5-methyltetrahydrofolate.

### RESULTS AND DISCUSSION

### *Recovery of radioactivity in liver and faeces*

Quantitative analyses of livers showed that in animals in the control group 12·1% of the dose of 2-[$^{14}$C]-folic acid was recovered in the liver 24 h after administration of the dose (Table I). The recovery of radioactivity in livers of animals dosed with MTX was 4·3% after a dose of 10mg/kg body wt and 2·8% after a dose of 100mg/kg body wt. These differences are highly significant ($P < 0{\cdot}001$ in both groups). Similar results were obtained when animals were given 2-[$^{14}$C]-5-methyltetrahydrofolate 24 h after a dose of MTX (100mg/kg body wt). Animals in the control group retained 13·5% of the radioactivity in the liver 24 h after the dose and animals receiving MTX retained 5·5% of the dose ($P < 0{\cdot}001$). In all of these groups of animals the liver radioactivity, extracted to prevent breakdown of polyglutamates, showed a single peak chromatographing as a polyglutamate on both Sephadex G.15 and DEAE cellulose.

Quantitative analysis of faeces showed that MTX (100 mg/kg) decreased the uptake of the folates from the intestine ($P < 0{\cdot}01$ for animals dosed with 2-[$^{14}$C]-folic acid, $P < 0{\cdot}05$ for animals dosed with 5-[$^{14}$C]-methyltetrahydrofolate and $P < 0{\cdot}10$ for animals dosed with 2-[$^{14}$C]-5-methyltetrahydrofolate (Table II).

TABLE I.—*Percentage recovery of radioactivity in the livers of rats receiving oral doses of [$^{14}$C]-folates 24 h after oral doses of MTX. For controls, animals were dosed with water 24 h before receiving [$^{14}$C]-folate. Number of animals in brackets*

| Folate | Animals received doses of: Water (controls) | | | MTX (10 mg/kg body wt) | | | MTX (100 mg/kg body wt) | | |
|---|---|---|---|---|---|---|---|---|---|
| | Mean | S.e. | | Mean | S.e. | | Mean | S.e. | |
| 2-[$^{14}$C]-folic acid (76 μg/kg body wt) | 12·1 | 3·8 | (8) | 4·3 | 1·4 | (8) | 2·8 | 1·1 | (6) |
| 2-[$^{14}$C]-5-methyltetrahydrofolate (7 μg/kg body wt) | 13·5 | 0·8 | (2) | — | | | 5·5 | 1·5 | (4) |

TABLE II.—*Percentage recovery of radioactivity in the faeces of rats receiving oral doses of [$^{14}$C]-folate 24 h after oral doses of MTX. Control animals were dosed orally with water 24 h before receiving [$^{14}$C]-folate. Number of animals in brackets*

| Folate | Animals received doses of: Water (controls) Mean | S.e. | | MTX 10 mg/kg body wt Mean | S.e. | | MTX 100 mg/kg body wt Mean | S.e. | |
|---|---|---|---|---|---|---|---|---|---|
| 2-[$^{14}$C]-folic acid (76 μg/kg body wt) | 36·3 | 7·9 | (8) | 42·5 | 12·6 | (8) | 48·4 | 4·8 | (6) |
| 5-[$^{14}$C]-5-methyltetrahydrofolate (70 μg/kg body wt) | 23·9 | 3·7 | (4) | — | | | 33·0 | 1·8 | (4) |
| 2-[$^{14}$C]-5-methyltetrahydrofolate (7 μg/kg body wt) | 34·4 | 4·5 | (4) | — | | | 38·4 | 2·2 | (6) |

TABLE III.—*Percentage recovery of dosed radioactivity in urine samples of rats receiving oral doses of [$^{14}$C]-folates 24 h after oral doses of MTX. Control animals were dosed with water 24 h before the dose of [$^{14}$C]-folates. For details see text. Number of animals in brackets*

| Folate | Animals received doses of: Water (controls) | | | | MTX 10 mg/kg body wt | | | | MTX 100 mg/kg body wt | | | |
|---|---|---|---|---|---|---|---|---|---|---|---|---|
| | 0–6 h | | 6–24 h | | 0–6 h | | 6–24 h | | 0–6 h | | 6–24 h | |
| | Mean | S.e. | Mean | S.e. | Mean | S.e. | Mean | S.e. | Mean | S.e. | Mean | S.e. |
| 2-[$^{14}$C]-folic acid (76 μg/kg body wt) | 8·4 | 1·8 | 9·9 | 2·8 | 22·2 | 2·6 | 9·2 | 2·3 | 21·2 | 2·6 | 11·8 | 2·3 |
| | (8) | | | | (8) | | | | (6) | | | |
| | 0–24 h | | | | | | | | 0–24 h | | | |
| | Mean | S.e. | | | | | | | Mean | S.e. | | |
| 2-[$^{14}$C]-5-methyltetrahydrofolate (7 μg/kg body wt) | 17·4 | 1·2 | | | — | | | | 19·8 | 0·8 | | |
| | (4) | | | | | | | | (6) | | | |

*Recovery of radioactivity in urine samples*

The recovery of radioactivity in urine samples over the 24h period is shown in Table III. Control animals excrete 8·5% of a dose of 2-[$^{14}$C]-folic acid in urine in 6 h. When 2-[$^{14}$C]-folic acid is given after MTX significantly more radioactivity is excreted in urine in the first 6 h after administration of the dose. The urinary excretion of radioactivity being 22·2% of the dose ($P < 0{\cdot}001$) after a dose of MTX of 10 mg/kg body wt. Much of this increase in urinary radioactivity is due to excretion of unmetabolized folic acid (Table IV) presumably caused by inhibition of dihydrofolate reductase. Also, animals that have been dosed with MTX excrete less of the urinary folates as 5-methyltetrahydrofolates.

There was no significant difference in recovery of radioactivity in urine and in urinary metabolites between normal rats dosed with 2-[$^{14}$C]-5-methyltetrahydrofolate and rats dosed with 2-[$^{14}$C]-5-methyltetrahydrofolate 24 h after a dose of MTX. In both groups of animals the major urinary folate was 5-methyltetra-

TABLE IV.—*Radioactivity in 24h urine samples associated with folic acid and 5-methyltetrahydrofolate in control animals and animals receiving oral doses of MTX (10 mg and 100 mg/kg body wt). For details see text. For numbers of animals see Table III*

| Animals | Total in urine. % of dose | Folic acid. % of dose | 5 Methyl-tetrahydra-folate. % of dose |
|---|---|---|---|
| Controls | 18·3 | 7·7 | 4·4 |
| MTX 10 mg. | 31·4 | 20·6 | 2·0 |
| MTX 100 mg. | 33·0 | 29·0 | 0·6 |

hydrofolate. When animals were given a mixture of 2-[$^{14}$C]-folic acid and 3′,5′,7,9-[$^{3}$H]-folic acid both $^{3}$H and $^{14}$C were recovered in urine samples. Quantitative analysis showed that there was no significant difference between the recovery of $^{3}$H and $^{14}$C in these urine samples, but chromatography of urine samples showed the presence of a metabolite labelled with $^{3}$H only that chromatographed with p-aminobenzoate or p-acetamidobenzoate (Connor *et al.*, 1979). Analysis of the urine figures showed that the excess of $^{3}$H over $^{14}$C in the fractions associated with reduced folate was increased in animals dosed with MTX (Table V).

TABLE V.—*Percentage recovery of radioactivity in 0–24h urine samples associated with non-folic acid peaks*

| | % radioactivity in urine less folic acid peak | | % $^{3}$H over $^{14}$C |
|---|---|---|---|
| | $^{3}$H | $^{14}$C | |
| Controls | 13·9 | 7·9 | 76 |
| MTX 10 mg/kg body wt | 17·6 | 8·8 | 100 |
| MTX 100 mg/kg body wt | 10·4 | 4·0 | 160 |

MTX decreases the amount of folate polyglutamate retained in rat livers when animals are dosed with 2-[$^{14}$C]-folic acid or 2-[$^{14}$]-5-methyltetrahydrofolate 24 h after being dosed with MTX. There are several possible explanations for this decrease in liver polyglutamate. The blood levels of the radioactive folate may be decreased because of decreased absorption of the label from the intestine. MTX may be decreasing the uptake of radiolabel into the liver, or it could cause decreased retention in the liver because the folate that entered the liver was metabolized less well to polyglutamate, or polyglutamate catabolism was increased. Lei *et al.* (1977) and Lucas *et al.* (1978) have shown that MTX does not affect folic acid uptake from the intestine *in vitro*, but has a marked effect if given 24 h before removal of the intestine for *in vitro* experiments. That decreased intestinal absorption could contribute to the reduced liver folate polyglutamate formation observed in these experiments is supported by the increased faecal recovery of radioactivity after oral 2-[$^{14}$C]-folic acid, 2-[$^{14}$C]-5-methyltetrahydrofolate and 5-[$^{14}$C]-5-methyltetrahydrofolate (Table II).

McGuire *et al.* (1979) have shown that tetrahydrofolate is a substrate for pteroylpolyglutamate synthetase while folic acid is not. The inability of the liver to convert large quantities of 2-[$^{14}$C]-folic acid to tetrahydrofolate and thus to polyglutamate may explain the decreased retention of radioactivity in the liver when radiolabelled folic acid is given after a dose of MTX. McBurney & Whitmore (1974) have suggested that conversion to polyglutamates serves to trap the folate monoglutamates that are transported into cells. MTX also decreases the retention of radioactivity in the liver when given 24 h before a dose of 2-[$^{14}$C]-5-methyltetrahydrofolate, which is a poor substrate for pteroylpolyglutamate synthetase (McGuire *et al.*, 1979). 5-methyltetrahydrofolate must lose the methyl group *in vivo* before it can be converted to a polyglutamate (Lavoie *et al.*, 1974), but no reduction step is necessary. MTX could reduce the levels of liver polyglutamate after a dose of 2-[$^{14}$C]-5-methyltetrahydrofolate at a step other than inhibiting the reduction of dihydrofolate reductase, probably by inhibiting liver folate transport, or by accelerating the catabolism of liver folate polyglutamates (Connor *et al.*, 1979) or both. That folate catabolism can occur after MTX is shown by the scission products after administration of mixed $^{3}$H- and $^{14}$C-labelled folic acid (Barford *et al.*, 1977). The results presented in this paper demonstrate an increase in scission after MTX administration, possibly by inhibiting reduction of dihydrofolate to tetrahydrofolate by dihydrofolate reductase, or by inhibition of dihydropteridine reductase, an enzyme which may maintain tetrahydrofolates in the fully reduced state (Pollock & Kaufman, 1978). Barford & Blair (1978) demonstrated a decrease in scission of the folate molecule in animals with a Walker 256 tumour.

The urinary folate results demonstrate that less of the dose of radiolabelled folic acid enters the folate pool in animals that have been dosed with MTX, but that more of the folate that enters the pool undergoes scission. The large excretion of folic acid is most probably due to failure of the radiolabelled folic acid to enter the folate pool because of inhibition of dihydrofolate reductase by MTX. There is no significant difference in urinary recovery of radioactivity between control animals and animals dosed with MTX when animals are given 2-[$^{14}$C]-5-methyltetrahydrofolate. Animals that have been dosed with 2-[$^{14}$C]-folic acid excrete both 5-methyltetrahydrofolate and 10-formylfolates in urine samples.

The results presented here demonstrate that MTX affects the uptake and retention of labelled oral folates in the whole animal, and reduces the size of the liver folate polyglutamate pool which has an essential primary coenzyme function in purine and pyrimidine biosynthesis (Rowe, 1978) and thus the therapeutic effect may not be due solely to inhibition of dihydrofolate reductase, but also to changes in distribution of folates in the animal. This paper describes for the first time a reduction in incorporation of folates in the whole animal into an essential coenzyme pool by the action of MTX, and demonstrates an increase in scission of the folate molecule after MTX administration.

The authors are grateful to the Cancer Research Campaign for financial support and to the Royal Society for a liquid scintillation counter.

### REFERENCES

BARFORD, P. A. & BLAIR, J. A. (1978) Effect of an implanted Walker tumour on metabolism of folic acid in the rat. *Br. J. Cancer*, **38**, 122.

BARFORD, P. A., STAFF, F. J. & BLAIR, J. A. (1977) Retained folates in the rat. *Biochem. J.*, **164**, 601.

BARFORD, P. A., STAFF, F. J. & BLAIR, J. A. (1978) The metabolic fate of 2- $^{14}$C-folic acid and a mixture of 2-$^{14}$C and 3′,5′,9-$^{3}$H folic acid in the rat. *Biochem. J.*, **174**, 579.

BARFORD, P. A., BLAIR, J. A. & MALGHANI, M. A. K. (1976) The effect of methotrexate on the metabolism of $^{14}$C-folates in the rat. *Biochem. Soc. Trans.*, **4**, 912.

BERTINO, J. R., PERKINS, J. P. & JOHNS, D. G. (1965) Purification and properties of dihydrofolate reductase from Erlich Ascites carcinoma cells. *Biochemistry*, **4**, 839.

CONNOR, M. J., PHEASANT, A. E. & BLAIR, J. A. (1979) The identification of p-acetamidobenzoate as a folate degradation product in rat urine. *Biochem. J.*, **178**, 795.

CHABNER, B. A., MYER, C. E., COLEMAN, C. N. & JOHNS, D. G. (1975) The clinical pharmacology of antineoplastic agents. *N. Engl. J. Med.*, **292**, 1107.

GOLDMAN, D. (1977) Effects of methotrexate on cellular metabolism: some critical elements in the drug–cell interaction. *Cancer Treatment Rep.*, **61**, 549.

HENDERSON, E. S., ADAMSON, R. H., DENHAM, C. & OLIVIERO, J. T. (1965*a*) The metabolic fate of tritiated methotrexate. I. Absorption, excretion and distribution in mice, rats, dogs and monkeys. *Cancer Res.*, **25**, 1008.

HENDERSON, E. S., ADAMSON, R. H. & OLIVIERO, J. T. (1965*b*) The metabolic fate of tritiated methotrexate. II. Absorption, excretion and distribution in man. *Cancer Res.*, **25**, 1018.

JOHNS, D. G. & BERTINO, J. R. (1973) Folate antagonists. In *Cancer Medicine* Eds Holland & Frei. Philadelphia: Lea & Febiger. 1st edition, p. 739.

LAVOIE, A., TRIPP, E. & HOFFBRAND, A. V. (1974) The effect of vitamin $B_{12}$ deficiency on methylfolate metabolism and pteroylpolyglutamate synthesis in human cells. *Clin. Sci. Mol. Med.*, **47**, 617.

LEI, F. H., LUCAS, M. L. & BLAIR, J. A. (1977) The influence of pH, low sodium ion concentration and methotrexate on the jejunal-surface pH: A model for folic acid transfer. *Biochem. Soc. Trans.*, **5**, 149.

LUCAS, M. L., SWANSTON, S. K., LEI, F. H., MANGKORNTHONG, P. & BLAIR, J. A. (1978) Effect of ethanol, diphenylhydantoin, methotrexate and low sodium ion concentration on jejunal-surface pH and folic acid transfer in the rat. *Biochem. Soc. Trans.*, **6**, 297.

MCBURNEY, M. W. & WHITMORE, G. F. (1974) Isolation and biochemical characterisation of folate deficient mutants of Chinese hamster cells. *Cell*, **2**, 173.

MCGUIRE, J. J., KITAMOTO, Y., HSIAH, P., COWARD, J. K. & BERTINO, J. R. (1979) Characterisation of mammalian polyglutamate synthetases. In *Chemistry and Biology of Pteridines*. Eds Kishick & Brown. North Holland: Elsevier. p. 471.

POLLOCK, R. J. & KAUFMAN, S. (1978) Dihydropteridine reductase may function in folate metabolism. *J. Neurochem.*, **31**, 115.

ROWE, P. B. (1978) In *The Metabolic Basis of Inherited Disease*. Eds. Stanbury, Wyngaarden & Frederickson. 4th edn. New York: McGraw-Hill. p. 430.